# Complete response to comprehensive treatment of a primary hepatic diffuse large B cell lymphoma: A case report

**DOI:** 10.3892/ol.2015.2920

**Published:** 2015-01-29

**Authors:** KAI JING ZHANG, SAI CHEN, JIAN LIN CHEN, LI HUA DONG

**Affiliations:** 1 Department of Hematology, Taizhou Central Hospital, Taizhou, Zhejiang 380000, P.R. China; 2 Department of Hematology, Henan Provincial Tumor Hospital, The Affiliated Cancer Hospital of Zhengzhou University, Zhengzhou, Henan 450008, P.R. China

**Keywords:** primary hepatic lymphoma, non-Hodgkin’s lymphoma, rituximab, chemotherapy

## Abstract

Primary hepatic lymphoma (PHL) is an uncommon lymphoid tumor with varied clinical features and treatment outcome. In the present study, the case of a 56-year-old patient with PHL and no clinical presentation was reported. During a routine physical examination, multiple hypodense nodules were incidentally detected in right lobes of the liver and hepatic portal in an abdominal computed tomography scan. A liver biopsy revealed the presence of a non-Hodgkin’s lymphoma diffuse large B cell type that was CD20-positive, followed by the diagnosis of a PHL. The patient was treated with R-CHOP, radiotherapy and R-Hyper-CVAD/R-HD MTX-ara-C, and complete remission was achieved.

## Introduction

Primary hepatic lymphoma (PHL) is a rare malignancy with nonspecific clinical features. PHL is defined by liver involvement at presentation; however, it does not affect the spleen, lymph nodes, peripheral blood, bone marrow or other tissues for at least six months following diagnosis (1). Main syptoms that are often exhibited include hepatic abnormalities in asymptomatic patients leading to the onset of fulminant hepatic failure with the rapid progression of encephalopathy leading to a comatose state and death. Hepatomegaly is also very common, and symptoms of jaundice may be found upon physical examination. Symptoms are usually nonspecific, and may include right upper quadrant and epigastric pain, fatigue, weight loss, fever, anorexia and nausea (2). PHL may occur at any age, the median age of PHL occurrence is 50 years, with a male to female ratio of 2–3:1 (3–5). Previous studies have indicated that the prevalence of PHL was 0.4% among extranodal NHL cases and 0.016% among all NHL cases (6,7). The etiology of PHL remains unclear, however, certain viruses, including hepatitis C virus (HCV), human immunodeficiency virus (HIV) and Epstein-Barr virus (EBV) may be involved. HCV infection is detected in 40–60% of patients with PHL (8–10). The pathogenesis of PHL remains unclear and may be associated with hepatitis C virus, human immunodeficiency virus, epstein-barr virus, or human T-lymphotropic virus infections, liver cirrhosis, systemic lupus erythematosus, and immunosuppressive therapy (8). To date, no optimal treatment method exists for PHL due to the rarity of this malignancy. However, surgical resectioning, radiotherapy and chemotherapy are currently available as treatment modalities. In the present study, the characteristics of PHL and a potential treatment strategy were evaluated in a patient presenting PHL. Written informed consent was obtained from the patient.

## Case report

In April 2006, an abdominal computed tomography (CT) scan revealed multiple solid hypodense lesions with a size of 18×18 mm in a 56-year-old male undergoing a routine physical examination at The Affiliated Cancer Hospital of Zhengzhou University (Zhengzhou, China). After four months, further CT scans detected an irregular nodular mass with a size of at least 125×100 mm. The patient did not present any symptoms, such as fever, respiratory problems, weakness, anorexia or weight loss. The patient had a 20-year history of hepatitis B virus infection and had been receiving treatment with lamivudine.

**Figure 1:**
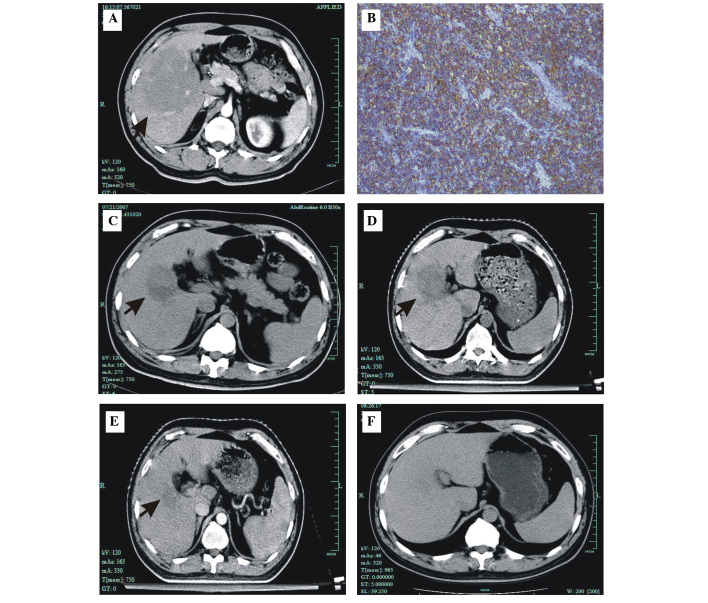
Abdominal CT scans at various times during the treatment: (A) Upon diagnosis, detecting a large mass lesion on the right liver and porta hepatis; (B) after two cycles of R-CHOP, showing a marked regression of the hepatic lesion; (C) after four cycles of R-CHOP, showing a slight increase of the hepatic lesion size compared with the size after two cycles; (D) after radiotherapy, showing further regression of hepatic lesion; and (E) after three cycles of R-HyperCVAD/R-MA, showing no hepatic lesion. (F) Immunohistochemical analysis showing CD20^+^ diffuse large B-cell lymphoma (stain, hematoxylin and eosin).

Clinical examination detected hepatomegaly (45 mm) below the right costal margin, while no other abnormalities were observed. The liver was nontender, with an irregular surface on the right side. Viral markers were found to be positive for hepatitis B and negative for hepatitis C. Fluorescence *in situ* hybridization revealed EBV-DNA levels of 5×10^6^ copies/ml. The results of further examinations, including a baseline electrocardiogram, chest CT scan, complete blood count, liver function test and kidney function test, were found to be normal. In addition, the levels of lactase dehydrogenase, uric acid, calcium, α-fetoprotein, β-2-microglobulin and carcinoma embryonic antigen were normal. An abdominal and pelvic CT scan revealed an irregular nodular mass in the right lobe of the liver with a size of at least 125×100 mm (Fig. 1A). No other abnormal findings were detected at the abdomen and pelvis. Furthermore, a chest and neck CT scan demonstrated normal lung fields and no mediastinal or paratracheal lymphadenopathy. Bone marrow biopsy identified no evidence of tumor involvement. Ultimately, a diagnostic liver biopsy was performed, which revealed sheets and nests of round cells, surrounded by mature lymphoid cells at the periphery. These cells were positive for the CD20 and leukocyte common antigen markers (Fig. 1B). The findings were consistent with non-Hodgkin’s lymphoma (NHL) of diffuse large B-cell type. Therefore, the final diagnosis of the patient in the present study was PHL of diffuse large B-cell type at stage IE, according to the Ann Arbor staging system, since a single extranodal site was involved (11).

The patient received R-CHOP chemotherapy, which involved administration of the following: 375 mg/m^2^ rituximab [intravenously (IV)] over 6 h on day –1; 750 mg/m^2^ cyclophosphamide over 3 h, 2 mg vincristine and 50 mg/m^2^ doxorubicin (IV) on day 1; and 40 mg/m^2^ prednisolone (orally) on days 1–5, every 21-day cycle. An abdominal CT scan was performed following two cycles of chemotherapy, which revealed a marked regression of the lesion to 58×58 mm in the right lobe of the liver (Fig. 1C). Following two further cycles of R-CHOP, an abdominal CT scan detected that the mass was slightly enlarged, having a size of 62×59 mm (Fig. 1D).

Subsequently, the right liver of the patient was treated with radiotherapy at a dose of 40 Gy over 28 days (2 Gy fractions, days 1–5). CT scans revealed further regression of the liver lesion to 26×32 mm following radiotherapy (Fig. 1E). In addition, the patient received treatment with the R-Hyper-CVAD/R-HD MTX-ara-C regimen, following radiotherapy. The regimen consisted of two cycles of dose-intensive therapy courses with rituximab and Hyper-CVAD (R-Hyper-CVAD) therapy alternating with rituximab and high-dose methotrexate (MTX) and cytosine arabinoside (ara-C) therapy (R-HD MTX-ara-C). The R-Hyper-CVAD regimen involved administration of the following: 375 mg/m^2^ rituximab (IV) on day –1; 300 mg/m^2^ cyclophosphamide (IV) over 3 h every 12 h for six doses on days 1–3; 300 mg/m^2^ mesna by continuous infusion initiated along with cyclophosphamide administration and ending 6 h after the last dose of cyclophosphamide; 2 mg vincristine (IV) on days 4 and 11; 50 mg/m^2^ doxorubicin (IV) on day 4; and 40 mg dexamethasone daily on days 1–4 and 11–14. The R-HD MTX-ara-C regimen involved administration of the following: 375 mg/m^2^ rituximab on day 1; 200 mg/m^2^ MTX (IV) over 2 h, followed by 800 mg/m^2^ IV over 24 h on day 1; 15 mg citrovorum factor rescue every 6 h, initiated 24 h after completion of MTX infusion and increased to 50 mg every 6 h when MTX levels were >20 mmol/l at the end of the infusion, >1 mmol/l at 24 h after infusion or >0.1 mmol/l at 48 h after infusion, until MTX levels were <0.1 mM; 3 g/m^2^ ara-C over 2 h every 12 h on days 2 and 3; and 50 mg methylprednisolone (IV) twice daily on days 1 through 3. After three cycles of the R-Hyper-CVAD/R-HD MTX-ara-C regimen, abdominal CT scans revealed that the liver lesion disappeared and the patient achieved complete remission (Fig. 1F). Following complete remission, the patient was subjected to maintenance therapy by administration of 375 mg/m^2^ rituximab on day 1 and every three months thereafter for a total of seven doses. The patient remained in a stable and healthy condition, with regular follow-ups for >3 years.

## Discussion

Non-Hodgkin’s lymphoma (NHL) is a common malignant disease, which presents with liver involvement in 10% of patients at an advanced stage of the disease (12). Primary hepatic lymphoma (PHL) refers to an extranodal lymphoma of the liver without involvement of any other organ, such as the lymph nodes or spleen (1). PHL is an unusual form of NHL that usually presents with constitutional symptoms, hepatomegaly and symptoms of cholestatic jaundice. Previous studies indicated that the prevalence of PHL was 0.4% among extranodal NHL cases and 0.016% among all the NHL cases (6,7). The etiology of PHL remains unknown; however, HCV and human immunodeficiency virus have been found to be implicated (7,8,13). HCV infection is detected in 40–60% of patients with PHL (8–10). The frequent association with HCV indicates that this virus may be involved in the pathogenesis of PHL (14,15). Furthermore, PHL is detected in immunocompromised patients; however, to the best of our knowledge, the association between PHL occurrence and immune deficiency has not been reported thus far. In the current study, the patient did not present HCV infection or immunodeficiency symptoms. The present study demonstrated that HBV or Epstein-Barr virus infection, which are important etiological factors for tumorigenesis, were a minor complication of the primary hepatic B-cell lymphoma case (8). Therefore, PHL may occur in patients without any prior liver disease.

To date, no consensus has been reached on the optimal treatment for PHL. Previous studies have reported the use of surgical resection, radiotherapy and chemotherapy as treatment modalities for PHL, alone or in combination (16–18). For low-volume localized PHL, surgical resection may be an effective treatment option, alone or in combination with chemotherapy (1,19,20). Since relapse following surgery is common and PHL is chemosensitive, chemotherapy may be employed in all the PHL cases. A study using transplantation combined chemotherapy conducted at the MD Anderson Cancer Center between 1974 and 1995 reported an overall complete remission rate of 83% and a five-year survival rate of 17% (21). Another study demonstrated that the five-year survival rate of primary hepatic diffuse large B-cell lymphoma (DLBCL) cases was 43%, while patients receiving rituximab along with cytotoxic treatment were successfully treated (19). In addition, a study from a group in France demonstrated that 24 HCV-seropositive systemic DLBCL cases exhibited a significantly lower two-year survival rate (56%) compared with 72 HCV-negative DLBCL cases (80%; P<0.02) (22). However, a good prognosis was observed in primary hepatic DLBCL cases following cytotoxic treatment with or without rituximab (23,24). In addition, a previous study revealed that antiviral treatment following cytotoxic treatment contributed towards a significantly increased disease-free survival period in 69 HCV-seropositive B-cell lymphoma cases (25). The use of the R-CHOP regimen has been found to increase the complete remission rate and prolong the survival significantly (17,24). These data strongly indicate that rituximab and cytotoxic treatment, followed by antiviral treatment, may result in an improved clinical outcome in primary hepatic and systemic HCV-seropositive DLBCL cases. The case reported in the present study was found to be sensitive to the R-CHOP regimen. However, resistance to the treatment was developed and the R-Hyper-CVAD/R-HD MTX-ara-C regimen was used in combination with radiotherapy. Complete remission was achieved in the patient. In conclusion, PHL is a rare disease and thus, at present no standard treatment has been identified. In the present case, the patient was treated with R-CHOP, radiotherapy and R-Hyper-CVAD/R-HD MTX-ara-C, and complete remission was achieved. Follow-up has shown that the patient remains in a stable condition. Thus, this case indicates that comprehensive treatment may be beneficial for patients with PHL.
